# Supplementary material for: Antarctic petrels ‘on the ice rocks’: wintering strategy of an Antarctic seabird
Source: R Soc Open Sci. 2020 Apr 1;7(4):191429. doi: 10.1098/rsos.191429 (PMC7211841; doi:10.1098/rsos.191429)
Supplement: Antarctic petrels ‘on the ice rocks’: wintering strategy of an Antarctic predator [file rsos191429supp1.docx]

**Supplementary material**

**Antarctic petrels ‘on the ice rocks’: wintering strategy of an Antarctic seabird**

K. Delord^1^, A. Kato^1^, A. Tarroux^2, 5^, F. Orgeret^1^, C. Cotté^3, 4^, Y. Ropert-Coudert^1^, Y. Cherel^1^, S. Descamps^5^

**Detailed Methods**

**Study species and tracking data**

Antarctic petrel is a circumpolar breeding Antarctic seabird [1,2]. We used tracking data from Antarctic petrels breeding at the world largest colony (Svarthamaren, Dronning Maud Land, Antarctica, 71°53’S, 5° 10’E), collected during the non-breeding season. Approximately 200,000 breeding pairs of Antarctic petrels breed in that colony, with large-inter-annual fluctuations [3]. Antarctic petrels feed by dipping and surface-seizing [4,5], primarily foraging on sea ice-associated fishes (*Pleuragramma antarctica* and *Electrona antarctica*) and krill (*Euphausia* spp.) [4]. Breeding adult Antarctic petrels from Svarthamaren, Dronning Maud Land were tagged during the 2011/2012 and 2012/2013 breeding seasons (hereafter 2012 and 2013) with LAT2500 geolocators (Lotek Wireless, Inc., Canada) and MK4083 geolocators (Biotrak Ltd., UK), and recaptured the following years. The geolocators also recorded saltwater immersion (wet/dry), providing data on birds’ activity (on water or on-land/flying). Activity was defined using different protocols according to the device type. Here, we use only data from Biotrack devices, as these record cumulative time spent in each activity and thus give a more complete image of the activity than Lotek devices, which record single data points recorded every minute (i.e. the time resolution is 20 times less than that of Biotrack GLS). Details on GLS data processing are provided in [3]. The utilization distributions (UDs) of 30, 50 and 95% were calculated using the ‘‘adehabitatHR’’ R package [6]. Residence time was defined as the proportion of time spent by each bird within a 2°×2° cell resolution. The 2° cell size was chosen on the basis of the mean accuracy of GLS locations (186 ± 114 km; [7] precluding analysis at smaller spatial scales. The point locations in each dataset were twice smoothed [7,8] and were converted into residence time distributions using time spent per cell (TSC). TSC-based methods have been extensively used to convert tracking data to gridded distributions [9–12]. Residence time was calculated using the trip-Grid function (*trip* package) in R [13], which creates a grid of time spent from each individual track by exact cell crossing methods, weighted by the time between locations for separate trip events, and then calculates the time spent in each cell. Then, we assigned the corresponding percentage of time spent in relation to the total trip duration. Finally, the TSC was converted to presence/absence gridded data and this was used in the subsequent analysis. The choice of the cell size was based on the mean accuracy of GLS locations (ca. 180 km) based on published studies [7,14].

**Environmental variables**

Sea ice contributes to an extensive proportion of the annual primary productivity in the Seasonal Ice Zone (SIZ) and is important in the life cycle of Antarctic krill [15] and pelagic fish, both primary food resources for Antarctic petrels [3,16]. Additionally, Antarctic petrels are known to forage widely within SIZ, where they target specific sea ice concentration areas [4,17].

Passive-microwave estimates of monthly sea-ice concentration (SIC) at 12.5 km resolution were obtained from the Institut Français de Recherche pour l'Exploitation de la Mer (<ftp://ftp.ifremer.fr/ifremer/cersat/products/gridded/psi-concentration/data/antarctic/monthly/netcdf/>). The widely used threshold in ice concentration of 15% has been used to define the ice edge [18]. Mean SIC for each 2° grid cell was calculated. ﻿

A monthly dataset of small icebergs (<3 km in length) is obtained from Iceberg Database of the Merged Altimeter for Altiberg project for 1992–2014 (<ftp://ftp.ifremer.fr/ifremer/cersat/projects/altiberg/>, [19]. The data are generated based on the analysis of high-resolution altimeter waveforms from images of nine satellite-based altimeters [20]. We used the surface area among the three available variables (iceberg presence probability, surface area, and volume) [21]. The dataset covers the Southern Ocean south of 40°S at a spatial resolution of 1°.

The Brigham Young University (BYU) Center for Remote Sensing produces and maintains an Antarctica Iceberg Tracking Database (http://www.scp.byu.edu/data/iceberg/database1.html) for icebergs longer than 5 km (hereafter named ‘large icebergs’) [22] routinely tracked and monitored since 1992, using six different satellite scatterometer instruments. The dataset contains the daily location for all identified icebergs. We extracted the track datasets and calculated the time spent per 2 degree cell.

SSH (Absolute dynamic topography) data is the Ssalto/Duacs altimeter product produced and distributed by the Copernicus Marine and Environment Monitoring Service (CMEMS) (http://www.marine.copernicus.eu) in 1/4 degree grid. According the contour methods [23], regions of high SSH gradient (that define a strong geostrophic current associated to fronts) are often collocated with a unique value of SSH. We have thus used SSH values to define fronts, particularly the Polar Front, as defined in [24].

Data was processed using R Core Team [13] and the package “*raster*” [25].

**Modelling presence**

We tested the influence of selected variables on the presence of birds. The dataset consisted of n=64 individual tracks (16982 locations) for which TSC (time spent per cell) was calculated. Residual normality was visually verified. A generalised additive mixed-effects model (GAMMs) with a binomial family and logit link function for presence/absence data (2009). Models were fit with R 3.4.1 [13].

Multi-collinearity among covariates was assessed using variance inflation factors (GVIFs, *AEDForecasting* library in R [26]; A cut-off value of 3 was used to remove collinear variables.

The response variable was a binary factor (presence/absence; *mgcv* library in R [27,28] where each level represented the occurrence of birds. In order to take into account the hierarchical structure of the data we added a random effect for each individual in the model. Indeed smooths terms were fitted with cubic regression spline with extra-shrinkage to avoid over-fitting [27,28]. We added the years and months in the fixed part as a factor in order to take into account the variability between the two years of monitoring.

Therefore, our initial model was:

$${E\left( {TSC}_{i} \right)=\beta}_{0}+(Month i)+\left( {Year}_{i} \right){+ f}_{1}\left( {SIC}_{i} \right)+f_{2}\left( {SmallIceb}_{i} \right)+f_{3}\left( {LargeIceb}_{i} \right)+f_{4}\left( {SSH}_{i} \right)+\varepsilon_{i}+\delta_{i}$$

where *E(TSC_i_)* is the expected value of the petrels distribution (presence/absence) as a function of the predictors, *β_0_* is an intercept, *f_k_* _=_ _1,... 4_ are nonparametric smoothing functions, *ε_i_* and *δ_i_* are normally distributed random effects with mean zero and variance$\sigma_{\varepsilon}^{2}$ and $\sigma_{\delta}^{2}$ , respectively and.

We ran one model with all months included and another set of models broken down by month. In each of these cases, the starting models included all the main effects. The best candidate model (i.e. the model containing the most informative set of covariates) was selected based on the second-order corrected Akaike’s information criterion (AIC). A difference of more than 2 AIC units was taken to indicate strong support for the model with the lower AIC, and a difference of less than 2 was taken to suggest that competing models received a similar amount of support from the data [29]. In this case, all of the best candidate models were presented.

**Modelling distance to sea-ice edge and distance to colony**

Some covariates were correlated to each other (Table S1) and explanatory variables collinear were dropped for the modelling of distribution of petrels, specifically the distance to sea-ice edge and distance to colony. We tested whether these covariates varied according to years and month. The effects were tested using generalised linear mixed-effects models (GLMMs) with a quasi-Poisson family and identity link function for distance to sea-ice edge and distance to colony [26]. GLMMs were used to test differences between years and where differences were not statistically significant data were pooled. In order to take into account the individual variability, we added a random effect for each individual in the model. The model validation and model selection were processed following [26].

**Feather collection and stable isotopes**

Feather stable isotope values were determined after recapture of the birds carrying GLS. Feathers reflect the diet at the time they were grown, because keratin is inert after synthesis [30–32]. In Antarctic fulmarine petrels, body moult is a gradual process extending over at least four months. It begins during late incubation, but most body feathers grow in the weeks following the completion of breeding [33,34]. Since the precise timing of synthesis of a given body feather was not known, isotopic measurements were performed on four fully-grown feathers per bird. Hence, it is likely that most body feathers carried dietary information about the previous interbreeding period corresponding to the GLS tracking period. The isotopic method was validated in the Southern Ocean, with δ^15^N values of seabirds increasing with trophic level [35], and δ^13^C values indicating their latitudinal foraging habitats [36,37]. Based on feather δ^13^C isoscapes, -21.2 ‰ was considered to correspond to the estimated isotopic location of the Polar Front and δ^13^C values < -21.2 ‰ to Antarctic waters [37].

Prior to isotopic analysis, single body feathers were cleaned of surface lipids and contaminants using a 2:1 chloroform: methanol solution for 2 min, followed by two successive methanol rinses. They were then oven dried for 48 hr at 50°C. Every whole feather was homogenized by cutting it with scissors into small fragments and a subsample of ~0.3 mg was packed into tin containers for stable isotope analysis. The relative abundance of carbon and nitrogen isotopes were determined with a continuous flow mass spectrometer (Thermo Scientific Delta V Advantage) coupled to an elemental analyser (Thermo Scientific Flash EA 1112). Results are presented in the usual δ notation relative to Vienna PeeDee Belemnite and atmospheric N_2_ for δ^13^C and δ^15^N, respectively. Replicate measurements of internal laboratory standards (acetanilide) indicated measurement errors < 0.15 ‰ for both δ^13^C and δ^15^N values.

Note the isotopic reference values of the main prey eaten by the petrels in Figure 3 of the main text are taken from the literature and were measured in feathers of chicks from gentoo (*Pygoscelis papua*) and king (*Aptenodytes patagonica*) penguins ([63] and [64] in the main text). As such, these isotopic values may be influenced by differential metabolic processes from adults that are related to the necessity for the chicks to increase their body mass and size rapidly.

**Modelling effect of the moon on activity**

The behavior was also well known to be impacted by the moon in seabirds species, through the influence on their activity level at breeding colonies or on their activity at-sea during or outside the breeding period [38–41]. The lunar cycle is believed to strongly influence the vertical distribution of potential marine prey species by improving their accessibility. These loggers through the record of activity data permit to explore the influence of the moon at a finer temporal scale.

General linear mixed-effects models (*lme4 and nlme* libraries in R; models GLMM 1 to 6; see Table S6) were used to model the daily proportion of time spent dry during daytime or darkness during the wintering period. Moon (lunar phase, normalized value of lunar phase or moon illumination) was included as fixed effects. The values of lunar phase (four phases: new, waxing, full and waning moon) and normalized value of lunar phase (value of lunar phase*100)/maximum value of lunar phase= 6.24537) were obtained using *lunar* library in R. The values of moon illumination were obtained using *suncalc* library in R. For all models, the variable "individual" was used as a random effect to account for pseudo-replication issues, since the same individual could provide several values. Response variables were visually tested for normality (through Q-Q plots) and homoscedasticity (following [26,42] before each statistical test.

Table S1. Testing for collinearity between different covariates before and after selection. VIF = variance inflation factor.

| Covariate | VIF test value  before variable selection | VIF test value  after variable selection |
| --- | --- | --- |
| Residence time of petrels | 1.04 | 1.02 |
| Longitude | 1.57 | 1.01 |
| Latitude | 18.66 | Not selected |
| Year | 1.00 | 1.00 |
| Month | 2.00 | 1.03 |
| Surface area of small icebergs | 1.12 | 1.08 |
| Residence time of large icebergs | 1.06 | 1.04 |
| Sea-ice concentration | 2.33 | 1.52 |
| Distance to the colony | 5.05 | Not selected |
| Distance to the sea-ice edge | 31.01 | Not selected |
| Sea surface height | 7.24 | 1.59 |

**Additional results**

Table S2. Results of the GLMM model distance to ice-edge in Antarctic petrels (DistIce) as a function of month. Variables selected in the best model (final model: DistIce~Month, with a random factor ‘individuals’) and reference values is April. Results suggest that individuals tended to reduce their distance to sea-ice edge in May and to maintain it during the all core non-breeding period. The degrees of freedom were 6236 for each month.

|  | Estimate | Std. Error | t-value | Pr (>\|t\|) |
| --- | --- | --- | --- | --- |
| (Intercept) | 596.76 | 16.54 | 36.08 | < 0.001 |
| May | -181.61 | 14.82 | -12.25 | < 0.001 |
| June | -190.78 | 14.60 | -13.07 | < 0.001 |
| July | -212.43 | 14.69 | -14.46 | < 0.001 |
| August | 38.93 | 13.55 | 2.87 | 0.004 |

Table S3. Results of the GLMM model distance to colony in Antarctic petrels (DistCol) as a function of month (final model: DistCol ~Month+Year, with a random factor ‘individuals’) and reference values are April and year 2012. Results suggest that individuals tended to increase their distance to their breeding colony during the all non-breeding period, with shorter distances during the 2013 period. The degrees of freedom were 6235 for each month.

|  | Estimate | Std. Error | t-value | Pr (>\|t\|) |
| --- | --- | --- | --- | --- |
| (Intercept) | 1515.35 | 51.67 | 29.33 | < 0.001 |
| May | 288.63 | 18.19 | 15.87 | < 0.001 |
| June | 627.38 | 18.45 | 34.00 | < 0.001 |
| July | 748.65 | 18.97 | 39.47 | < 0.001 |
| August | 1031.25 | 17.49 | 58.97 | < 0.001 |
| 2013 | -271.41 | 53.36 | -5.09 | < 0.001 |

Table S4. Results of the GAMM model explaining (presence/absence) in Antarctic petrels as a function of environmental covariates during a) April, b) May, c) June, d) July and e) August. Variables selected in the best model (Table S3) and reference value residence during 2012.

| April | **Value** | **Std.Error** | **P-value** |
| --- | --- | --- | --- |
| (Intercept) | -58.23 | 30.39 | 0.055 |
| Year 2013 | 0.51 | 0.11 | < 0.001 |
|  | **Smoother edf** | **Chi.sq** | **P-value** |
| Small icebergs area | 1.87 | 4.69 | 0.125 |
| Large icebergs residence time | 1.00 | 3.09 | 0.079 |
| Sea ice concentration | 1.86 | 234.03 | < 0.001 |
| Sea surface height | 1.99 | 150.54 | < 0.001 |
| Bird ID | 30.08 | 103.06 | < 0.001 |

| May | **Value** | **Std.Error** | **P-value** |
| --- | --- | --- | --- |
| (Intercept) | -9.31 | 1.13 | < 0.001 |
| Year 2013 | 0.09 | 0.08 | 0.291 |
|  | **Smoother edf** | **Chi.sq** | **P-value** |
| Small icebergs area | 2.61 | 20.05 | < 0.001 |
| Large icebergs residence time | 1.00 | 12.91 | < 0.001 |
| Sea ice concentration | 3.79 | 259.14 | < 0.001 |
| Sea surface height | 2.58 | 608.50 | < 0.001 |
| Bird ID | 33.68 | 153.34 | < 0.001 |

| June | **Value** | **Std.Error** | **P-value** |
| --- | --- | --- | --- |
| (Intercept) | -6.79 | 0.22 | < 0.001 |
| Year 2013 | -0.15 | 0.09 | 0.09 |
|  | **Smoother edf** | **Chi.sq** | **P-value** |
| Small icebergs area | 3.26 | 140.13 | < 0.001 |
| Large icebergs residence time | 2.74 | 66.61 | < 0.001 |
| Sea ice concentration | 3.91 | 495.73 | < 0.001 |
| Sea surface height | 2.95 | 1109.87 | < 0.001 |
| Bird ID | 38.62 | 342.39 | < 0.001 |

| July | **Value** | **Std.Error** | **P-value** |
| --- | --- | --- | --- |
| (Intercept) | -7.31 | 0.28 | < 0.001 |
| Year 2013 | 0.02 | 0.09 | 0.842 |
|  | **Smoother edf** | **Chi.sq** | **P-value** |
| Large icebergs residence time | 3.89 | 118.4 | < 0.001 |
| Sea ice concentration | 3.17 | 609.6 | < 0.001 |
| Sea surface height | 3.78 | 1260.3 | < 0.001 |
| Bird ID | 38.10 | 376.1 | < 0.001 |

| August | **Value** | **Std.Error** | **P-value** |
| --- | --- | --- | --- |
| (Intercept) | -5.96 | 0.18 | < 0.001 |
| Year 2013 | 0.32 | 0.07 | < 0.001 |
|  | **Smoother edf** | **Chi.sq** | **P-value** |
| Small icebergs area | 2.84 | 26.87 | < 0.001 |
| Large icebergs residence time | 3.42 | 30.44 | < 0.001 |
| Sea ice concentration | 3.67 | 630.83 | < 0.001 |
| Sea surface height | 3.37 | 1524.59 | < 0.001 |
| Bird | 40.16 | 621.80 | < 0.001 |

Table S5. Detailed list of the analyses performed on activity data (Generalized Linear Mixed Models with a random factor ‘individual’).

| Analyse | Model number | Study variable | Final model | Sample size |
| --- | --- | --- | --- | --- |
| GLMM | 1 | Daily proportion of time spent dry during daytime, asin(sqrt()) | ~ lunar.4phase^1^ | 2777 |
| GLMM | 2 | Daily proportion of time spent dry during darkness, asin(sqrt()) | ~ lunar.4phase^1^ | 2777 |
| GLMM | 3 | Daily proportion of time spent dry during daytime, asin(sqrt()) | ~ lunar.n^2^ | 2777 |
| GLMM | 4 | Daily proportion of time spent dry during darkness, asin(sqrt()) | ~ lunar.n^2^ | 2777 |
| GLMM | 5 | Daily proportion of time spent dry during daytime, asin(sqrt()) | ~ visible.moon^3^ | 2777 |
| GLMM | 6 | Daily proportion of time spent dry during darkness, asin(sqrt()) | ~ visible.moon^3^ | 2777 |

^1^ four lunar phases: new, waxing, full and waning; ^2^ normalization of the lunar phase value; ^3^ moon illumination value

Table S6. Fixed-effect parameters of generalized linear mixed model of the variation of the activity pattern (daily proportion of time spent dry during daytime) for Antarctic petrel during the wintering period. Variables selected in the best model (GLMM1 see Table S6) and reference value is new moon (lunar phaseNew). The degrees of freedom were 2751 for each variable.

|  | Value | Std,Error | t-value | p-value |
| --- | --- | --- | --- | --- |
| **(Intercept)** | 0.945 | 0.02 | 42.83 | **0.000** |
| lunar.4phaseWaxing | 0.004 | 0.01 | 0.31 | 0.758 |
| lunar.4phaseFull | -0.023 | 0.01 | -1.60 | 0.111 |
| lunar.4phaseWaning | 0.018 | 0.01 | 1.29 | 0.199 |

Table S7. Fixed-effect parameters of generalized linear mixed model of the variation of the activity pattern (daily proportion of time spent dry during nighttime) for Antarctic petrel during the wintering period. Variables selected in the best model (GLMM2 see Table S6) and reference value is new moon (lunar phaseNew). The degrees of freedom were 2751 for each variable.

|  | Value | Std,Error | t-value | p-value |
| --- | --- | --- | --- | --- |
| **(Intercept)** | 1.147 | 0.02 | 51.37 | **0.000** |
| lunar.4phaseWaxing | -0.004 | 0.01 | -0.34 | 0.730 |
| lunar.4phaseFull | 0.003 | 0.01 | 0.29 | 0.772 |
| lunar.4phaseWaning | -0.015 | 0.01 | -1.29 | 0.196 |

Table S8. Fixed-effect parameters of generalized linear mixed model of the variation of the activity pattern (daily proportion of time spent dry during daytime) for Antarctic petrel during the wintering period. Variables selected in the best model (GLMM3 see Table S6). The degrees of freedom were 2753 for each variable.

|  | Value | Std,Error | t-value | p-value |
| --- | --- | --- | --- | --- |
| **(Intercept)** | 0.944 | 0.02 | 42.85 | **0.000** |
| lunar.n | 0.000 | 0.00 | 0.17 | 0.863 |

Table S9. Fixed-effect parameters of generalized linear mixed model of the variation of the activity pattern (daily proportion of time spent dry during darkness for Antarctic petrel during the wintering period. Variables selected in the best model (GLMM4 see Table S6). The degrees of freedom were 2753 for each variable.

|  | Value | Std,Error | t-value | p-value |
| --- | --- | --- | --- | --- |
| **(Intercept)** | 1.145 | 0.02 | 51.35 | **0.000** |
| lunar.n | -0.000 | 0.00 | -0.34 | 0.737 |

Table S10. Fixed-effect parameters of generalized linear mixed model of the variation of the activity pattern (daily proportion of time spent dry during daytime) for Antarctic petrel during the wintering period. Variables selected in the best model (GLMM5 see Table S6). The degrees of freedom were 2753 for each variable.

|  | Value | Std,Error | t-value | p-value |
| --- | --- | --- | --- | --- |
| **(Intercept)** | 0.958 | 0.02 | 45.05 | **0.000** |
| visible.moon | -0.026 | 0.01 | -1.81 | 0.071 |

Table S11. Fixed-effect parameters of generalized linear mixed model of the variation of the activity pattern (daily proportion of time spent dry during darkness) for Antarctic petrel during the wintering period. Variables selected in the best model (GLMM6 see Table S6). The degrees of freedom were 2753 for each variable.

|  | Value | Std,Error | t-value | p-value |
| --- | --- | --- | --- | --- |
| **(Intercept)** | 1.142 | 0.02 | 52.37 | **0.000** |
| visible.moon | 0.000 | 0.01 | 0.035 | 0.972 |

Figure S1. Proportion (± S.D.) of time Antarctic petrels spent in a dry state (out of water) according to lunar phase during daytime (open circles) and during nighttime (closed circles).


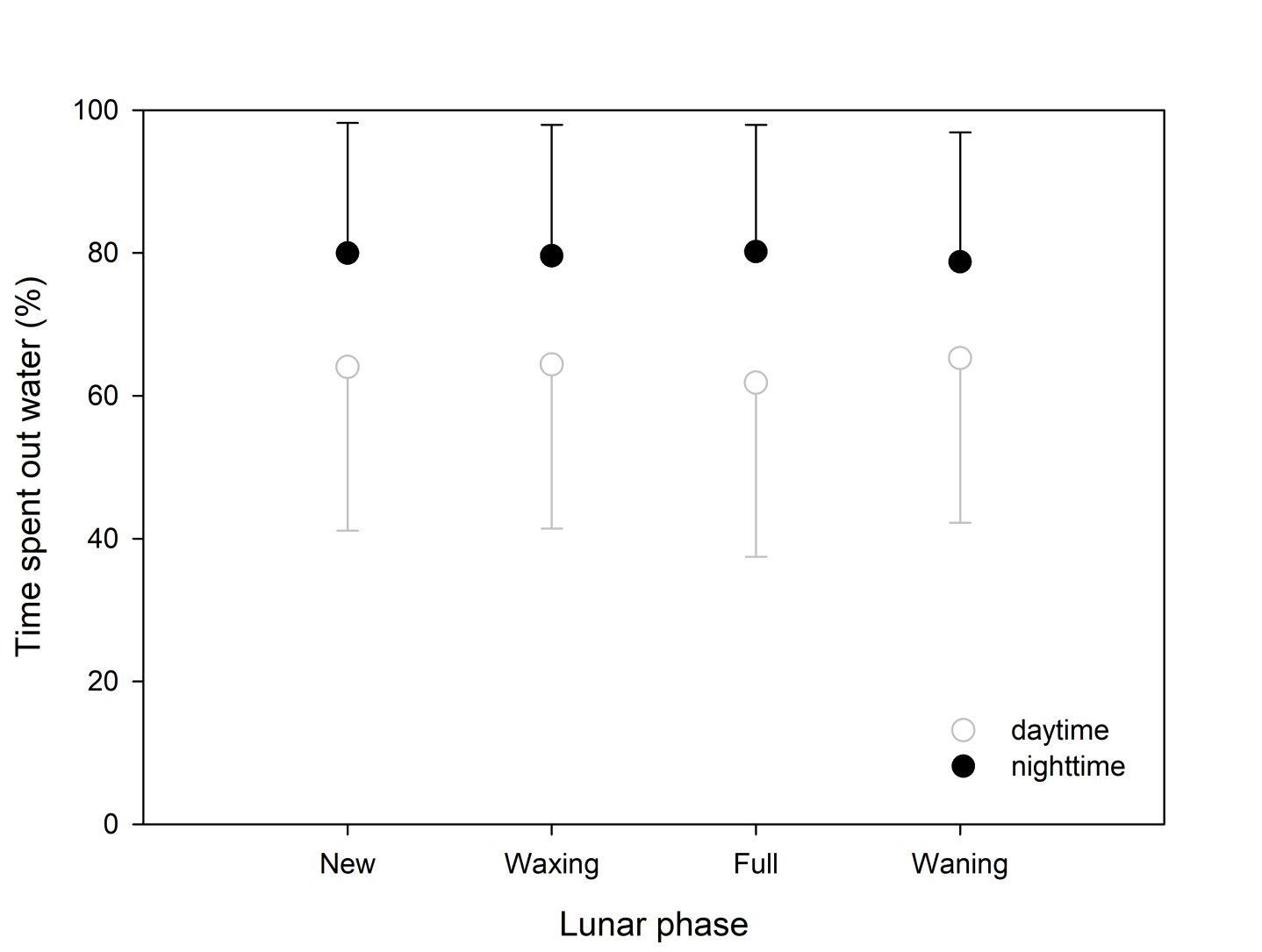


Figure S2. Estimated smoothing curves (with s.e.) for environmental covariates in relation with the presence probability of petrels during a) April, b) May, c) June, d) July and e) August. Covariates considered are small (SQ.ice) and large icebergs (SQ.liceberg), sea-ice concentration (SQ.SIC) and sea surface height (SSH). Interpretation of gamms results must be taken with caution where few observation data and large confidence interval exist.
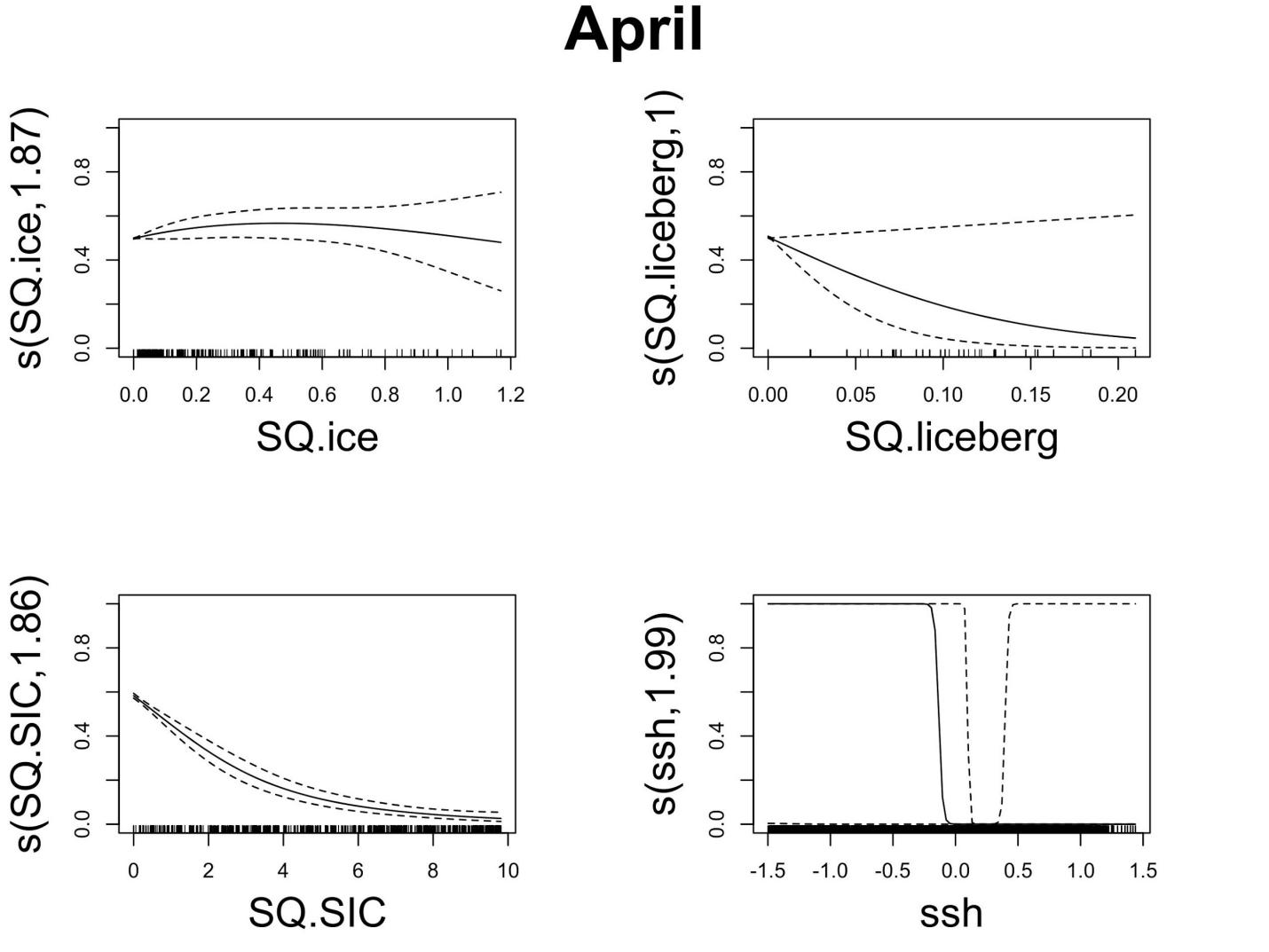

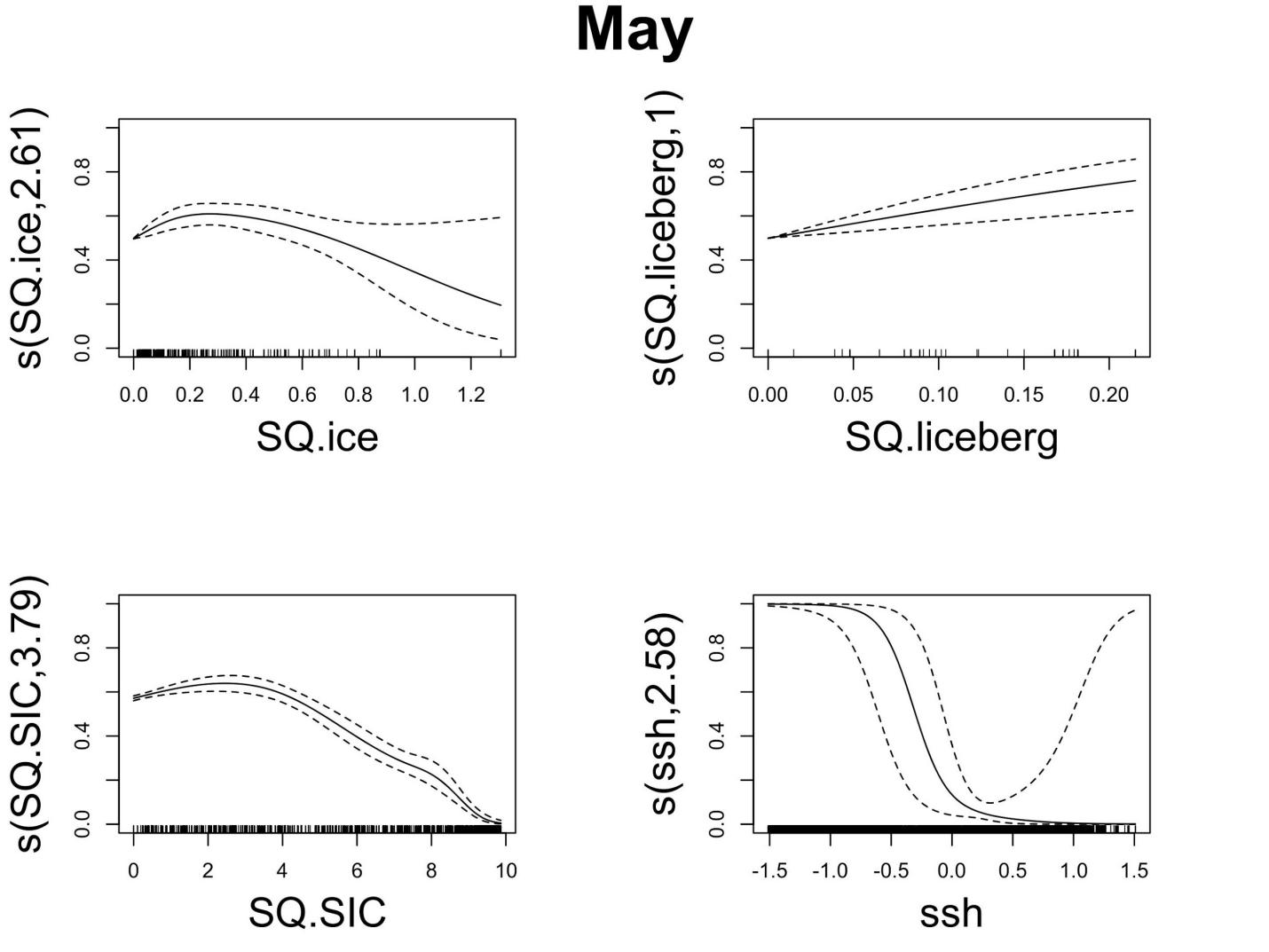


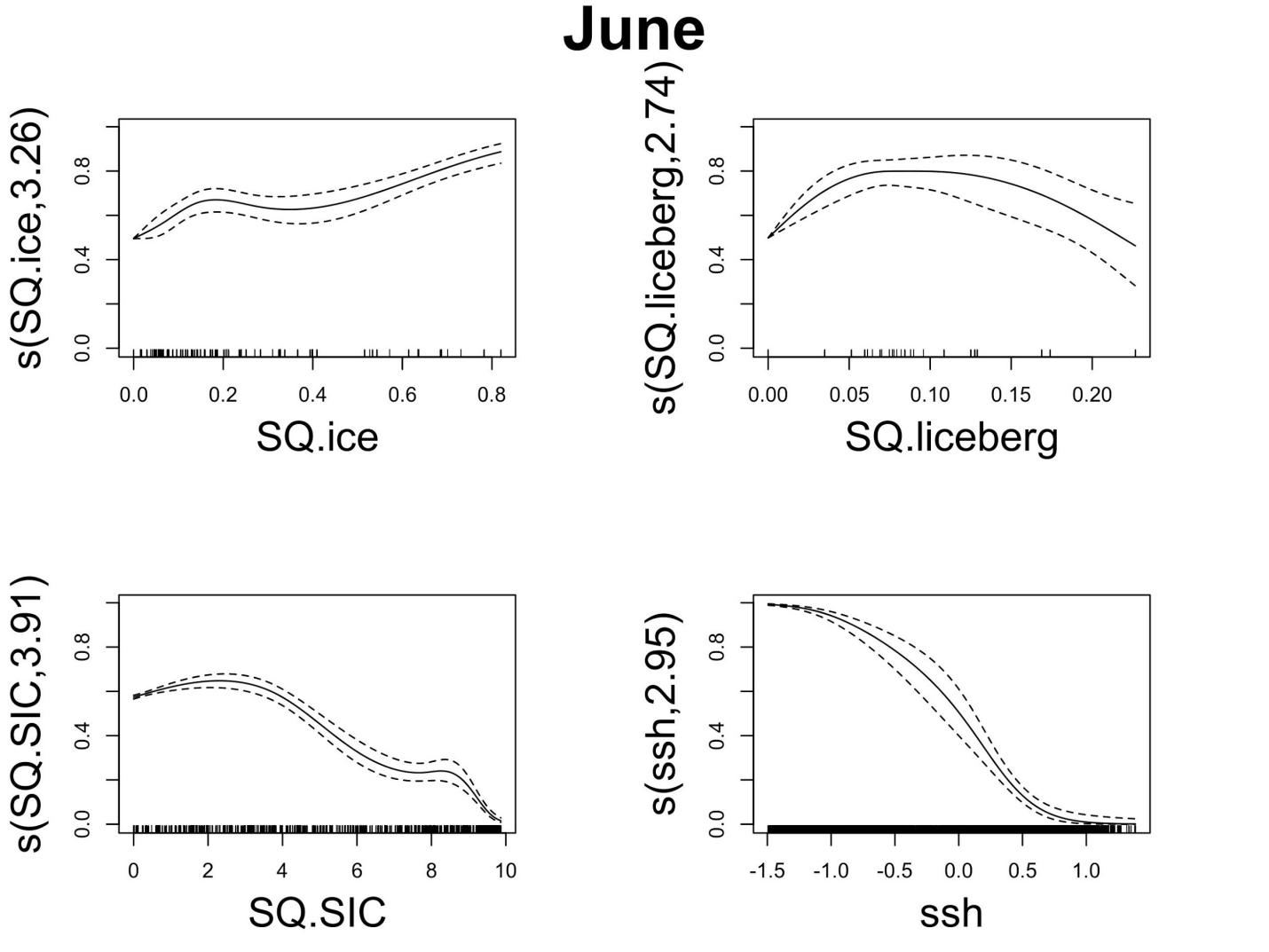

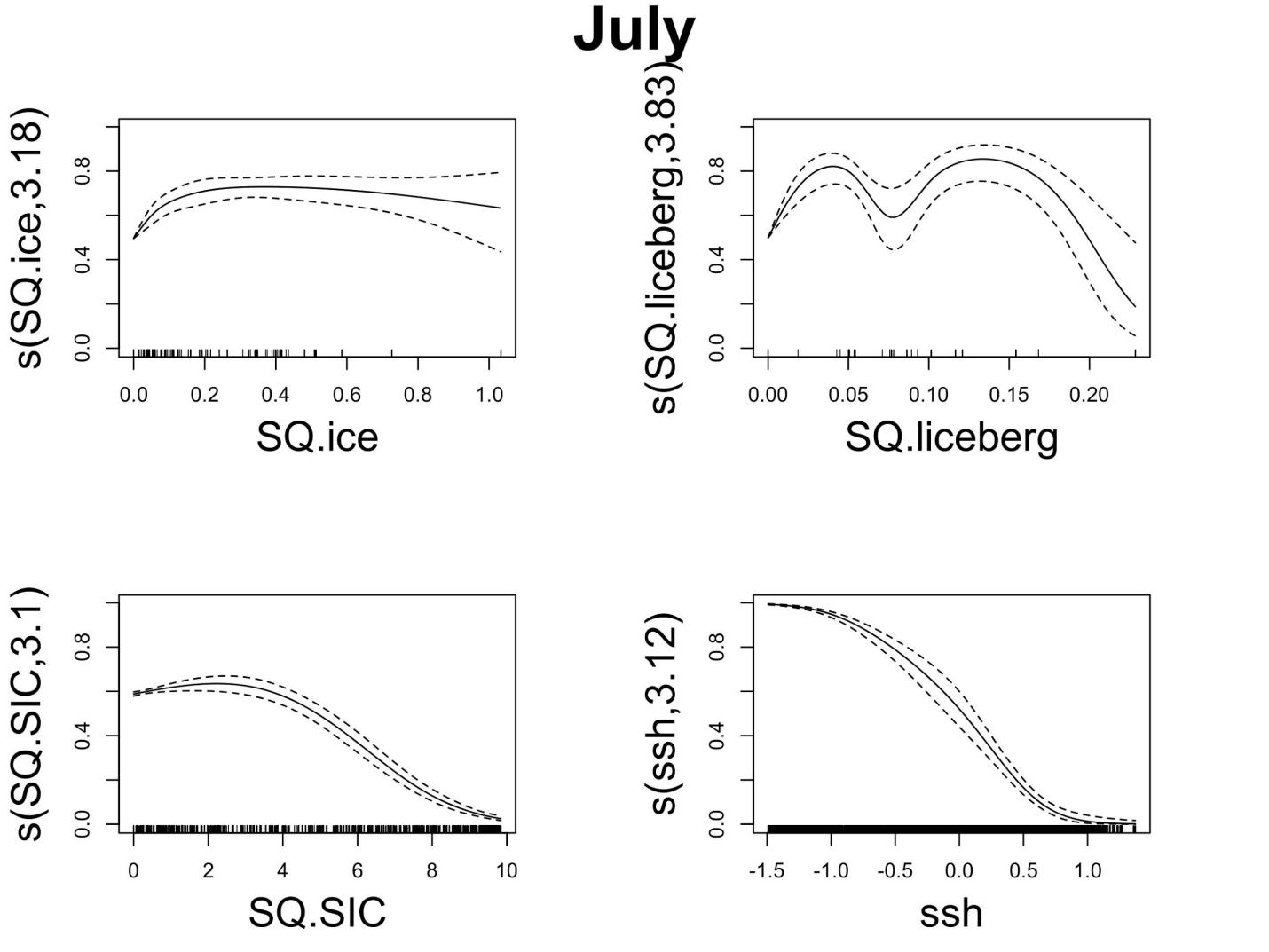

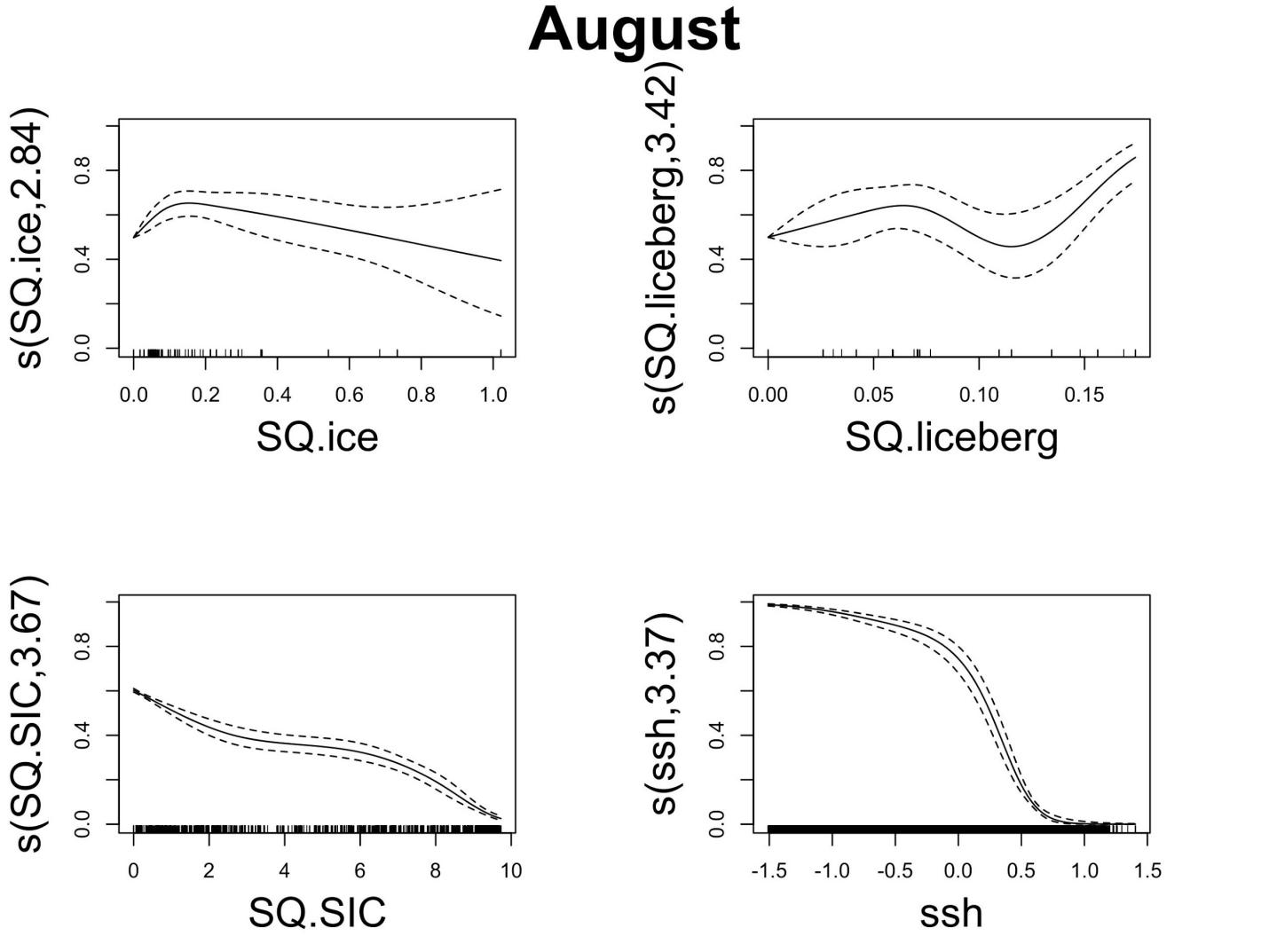


**References**

1. van Franeker JA, Gavrilo M, Mehlum F, Veit RR, Woehler EJ. 1999 Distribution and abundance of the Antarctic Petrel. *Waterbirds* **22**, 14–28. (doi:10.2307/1521989)

2. Hodum PJ. 2002 Breeding biology of high-latitude Antarctic fulmarine petrels (Procellariidae). *J. Zool.* **256**, 139–149.

3. Descamps S *et al.* 2016 At-Sea Distribution and Prey Selection of Antarctic Petrels and Commercial Krill Fisheries. *Plos One* **11**.

4. Ainley DG, O’Connor EF, Boekelheide RJ. 1984 The marine ecology of birds in the Ross Sea, Antarctica. *Ornithol. Monogr.* **32**, 1–97.

5. Harper PC, Croxall JP, Cooper J. 1985 A guide to foraging methods used by marine birds in antarctic and subantarctic seas. *Biomass Handb.* **24**, 1–22.

6. Calenge C. 2006 The package adehabitat for the R software: a tool for the analysis of space and habitat use by animals. *Ecol. Model.* **197**, 516–519.

7. Phillips RA, Silk JRD, Croxall JP, Afanasyev V, Briggs DR. 2004 Accuracy of geolocation estimates for flying seabirds. *Mar. Ecol.-Prog. Ser.* **266**, 265–272.

8. Catry I, Dias MP, Catry T, Afanasyev V, Fox J, Franco AM, Sutherland WJ. 2011 Individual variation in migratory movements and winter behaviour of Iberian Lesser Kestrels Falco naumanni revealed by geolocators. *Ibis* **153**, 154–164.

9. Louzao M, Pinaud D, Peron C, Delord K, Wiegand T, Weimerskirch H. 2011 Conserving pelagic habitats: seascape modelling of an oceanic top predator. *J. Appl. Ecol.* **48**, 121–132.

10. Pedersen MW, Patterson TA, Thygesen UH, Madsen H. 2011 Estimating animal behavior and residency from movement data. *Oikos* **120**, 1281–1290.

11. Péron C, Delord K, Phillips RA, Charbonnier Y, Marteau C, Louzao M, Weimerskirch H. 2010 Seasonal variation in oceanographic habitat and behaviour of white-chinned petrels *Procellaria aequinoctialis* from Kerguelen Island. *Mar. Ecol.-Prog. Ser.* **416**, 267–284.

12. Péron C, Weimerskirch H, Bost C-A. 2012 Projected poleward shift of king penguins’ (*Aptenodytes patagonicus*) foraging range at the Crozet Islands, southern Indian Ocean. *Proc. R. Soc. LondonSeries B Biol. Sci.* **279**, 2515–2523.

13. R Core Team. In press. R: A language and environment for statistical computing.

14. Merkel B, Phillips RA, Descamps S, Yoccoz NG, Moe B, Strom H. 2016 A probabilistic algorithm to process geolocation data. *Mov. Ecol.* **4**, 26. (doi:10.1186/s40462-016-0091-8)

15. Loeb V, Siegel V, Holm-Hansen O, Hewitt R, Fraser W, Trivelpiece W, Trivelpiece S. 1997 Effects of sea-ice extent and krill or salp dominance on the Antarctic food web. *Nature* **387**, 897. (doi:10.1038/43174)

16. Lorentsen SH, Klages N, Rov N. 1998 Diet and prey consumption of Antarctic petrels Thalassoica antarctica at Svarthamaren, Dronning Maud Land, and at sea outside the colony. *Polar Biol.* **19**, 414–420. (doi:10.1007/s003000050267)

17. Ainley DG, Ribic CA, Spear LB. 1993 Species-habitat relationships among Antarctic seabirds: a function of physical or biological factors? *Condor* , 806–816. (doi:10.2307/1369419)

18. Massom RA, Hill K, Barbraud C, Adams N, Ancel A, Emmerson L, Pook MJ. 2009 Fast ice distribution in Adelie Land, East Antarctica: interannual variability and implications for emperor penguins Aptenodytes forsteri. *Mar. Ecol. Prog. Ser.* **374**, 243–257. (doi:10.3354/meps07734)

19. Altiberg Project. In press. Altiberg Project: Iceberg Database of the Merged Altimeters.

20. Tournade J, Bouhier N, Girard-Ardhuin F, Rémy F. 2016 Antarctic icebergs distributions 1992-2014. *J. Geophys. Res.-Oceans* **120**, 1954–1974.

21. Tournade J, Girard-Ardhuin F, Legrésy B. 2012 Antarctic icebergs distributions, 2002-2010. *J. Geophys. Res.* **117**.

22. Stuart KM, Long DG. 2011 Iceberg size and orientation estimation using SeaWinds. *Cold Reg. Sci. Technol.* **69**, 39–51.

23. Sokolov S, Rintoul SR. 2002 Structure of Southern Ocean fronts at 140 E. *J. Mar. Syst.* **37**, 151–184.

24. Chapman CC. 2017 New perspectives on frontal variability in the Southern Ocean. *J. Phys. Oceanogr.* **47**, 1151–1168.

25. Hijmans R. 2018 raster: Geographic Data Analysis and Modeling. R package version 2.6-7.

26. Zuur AF, editor. 2009 *Mixed effects models and extensions in ecology with R*. New York, NY: Springer.

27. Wood S. 2004 Stable and efficient multiple smoothing parameter estimation for generalized additive models. *J. Am. Stat. Assoc.* **99**, 673–686.

28. Wood S. 2008 Fast stable direct fitting and smoothness selection for generalized additive models. *J. Am. Stat. Assoc.* **70**, 495–518.

29. Burnham KP, Anderson DR. 2002 *Model Selection and Multimodel Inference: A Practical Information-Theoretic Approach*. NewYork,NY.

30. Hobson KA, Clark RG. 1992 Assessing avian diets using stable isotopes I: turnover of ^13^C in tissues. *The Condor* **94**, 181–188.

31. Hobson KA, Clark RG. 1993 Turnover of ^13^C in cellular and plasma fractions of blood: implications for non destructive sampling in avian dietary studies. *The Auk* **110**, 638–641.

32. Bearhop S, Waldron S, Votier SC, Furness RW. 2002 Factors that influence assimilation rates and fractionation of nitrogen and carbon stable isotopes in avian blood and feathers. *Physiol. Biochem. Zool.* **75**, 451–458.

33. Beck J. 1970 Breeding seasons and moult in some smaller Antarctic petrels. *Antarct. Ecol.* **1**, 542–550.

34. Marchant S, Higgins PJ. 1990 *Handbook of Australian, New Zealand and Antarctic birds*. Oxford University Press, Melbourne.

35. Cherel Y, Fontaine C, Richard P, Labat JP. 2010 Isotopic niches and trophic levels of myctophid fishes and their predators in the Southern Ocean. *Limnol. Oceanogr.* **55**, 324–332.

36. Cherel Y, Hobson KA. 2007 Geographical variation in carbon stable isotope signatures of marine predators: a tool to investigate their foraging areas in the Southern Ocean. *Mar. Ecol. Prog. Ser.* **329**, 281–287.

37. Jaeger A, Lecomte VJ, Weimerskirch H, Richard P, Cherel Y. 2010 Seabird satellite tracking validates the use of latitudinal isoscapes to depict predators’ foraging areas in the Southern Ocean. *Rapid Commun. Mass Spectrom.* **24**, 3456–3460.

38. Yamamoto T, Takahashi A, Yoda K, Katsumata N, Watanabe S, Sato K, Trathan PN. 2008 The lunar cycle affects at-sea behaviour in a pelagic seabird, the streaked shearwater, Calonectris leucomelas. *Anim. Behav.* **76**, 1647–1652.

39. Dias MP, Romero J, Granadeiro JP, Catry T, Pollet IL, Catry P. 2016 Distribution and at-sea activity of a nocturnal seabird, the Bulwer’s petrel Bulweria bulwerii, during the incubation period. *Deep Sea Res. Part Oceanogr. Res. Pap.* **113**, 49–56.

40. Ramos R, Ramirez I, Paiva VH, Militao T, Biscoito M, Menezes D, Phillips RA, Zino F, Gonzalez-Solis J. 2016 Global spatial ecology of three closely-related gadfly petrels. *Sci. Rep.* **6**.

41. Waap S, Symondson WO, Granadeiro JP, Alonso H, Serra-Gonçalves C, Dias MP, Catry P. 2017 The diet of a nocturnal pelagic predator, the Bulwer’s petrel, across the lunar cycle. *Sci. Rep.* **7**, 1384.

42. Zuur AF, Ieno EN, Elphick CS. 2010 A protocol for data exploration to avoid common statistical problems. *Methods Ecol. Evol.* **1**, 3–14. (doi:10.1111/j.2041-210X.2009.00001.x)
